# Amino acid "little Big Bang": Representing amino acid substitution matrices as dot products of Euclidian vectors

**DOI:** 10.1186/1471-2105-11-4

**Published:** 2010-01-04

**Authors:** Karel Zimmermann, Jean-François Gibrat

**Affiliations:** 1 Université Pierre et Marie Curie (Paris VI), France; 2 INRA, Mathématique, Informatique et Génome UR1077, F-78352 Jouy-en-Josas, France

## Abstract

**Background:**

Sequence comparisons make use of a one-letter representation for amino acids, the necessary quantitative information being supplied by the substitution matrices. This paper deals with the problem of finding a representation that provides a comprehensive description of amino acid intrinsic properties consistent with the substitution matrices.

**Results:**

We present a Euclidian vector representation of the amino acids, obtained by the singular value decomposition of the substitution matrices. The substitution matrix entries correspond to the dot product of amino acid vectors. We apply this vector encoding to the study of the relative importance of various amino acid physicochemical properties upon the substitution matrices. We also characterize and compare the PAM and BLOSUM series substitution matrices.

**Conclusions:**

This vector encoding introduces a Euclidian metric in the amino acid space, consistent with substitution matrices. Such a numerical description of the amino acid is useful when intrinsic properties of amino acids are necessary, for instance, building sequence profiles or finding consensus sequences, using machine learning algorithms such as Support Vector Machine and Neural Networks algorithms.

## Background

Methods for analyzing protein sequences rest on the underlying amino acid representation. For many purposes, such as sequence comparisons, amino acids are represented by a one-letter code and their similarity is "summed up" in substitution (scoring) matrices.

Elements of these matrices represents the score of substituting an amino acid by another one in homologous proteins. It has been shown [1] that the general form of such an element is:

where *s*_*ab *_is the matrix element corresponding to amino acids *a *and *b*, *P*_*ab *_is the probability to find these amino acids aligned together in known protein families, and *P*_*a*_, *P*_*b *_are the corresponding background frequencies. *λ *is a scaling factor. PAM [2] and BLOSUM [3] matrices are computed accordingly. Such a ratio compare the probability of an event under two alternative hypotheses: i) the amino acids are aligned because the two sequences are evolutionary related or ii) the alignment is due to a chance occurrence. Adding such scores when comparing two sequences therefore amounts to maximizing the probability that the two sequences are evolutionary related.

Protein substitution matrices play a central role in sequence comparisons. They permit to align and compare quantitatively any two protein sequences, but they do not provide a description of the individual amino acids themselves.

Some analyses require taking into consideration the *intrinsic *properties of the sequences. For instance, this is the case of the prediction of signal peptide cleavage sites [4], disordered regions, low complexity zones, transmembrane segments, secondary structures, etc.

The idea of numerical encoding of the individual amino acids is not new. As far as we are aware, Swanson was the first to propose a representation of amino acids by vectors [5]. [4] addressed the issue of the best amino acid encoding to be used with machine learning algorithms. Some approaches are based on various properties of amino acids [5-8]. Other approaches start from the substitution matrices. However, with few exceptions, e.g., [9], most authors start by transforming them into distance matrices [4,10-14]. This approach is not devoid of difficulties. First, such a conversion is not unique and it is not known which one is the most appropriate, if any. Second, it is intuitively assumed that the distance is some inverse of the similarity, i.e., when the similarity is large the distance should be small and vice versa. However, the diagonal of a substitution matrix contains very different values (measuring the "mutatibility" of amino acids), but the diagonal of a distance matrix is always zero by the very definition of the distance. Despite these known difficulties, we will briefly allude to distance matrix approaches in the discussion.

Thus, on the one hand, there exist substitution matrices but no corresponding representation of individual amino acids and on the other hand there are various amino acid (vector) codings, which, at best, correspond very indirectly to any current substitution matrix.

This paper deals with the problem of finding amino acid Euclidian vectors corresponding to current substitution matrices. These vectors are obtained by the singular value decomposition of the matrices. The substitution matrix entries correspond to the dot product of the corresponding amino acids vectors. As an example of application of this representation, we study the significance of various amino acid physicochemical properties upon the corresponding substitution matrices.

## Methods

### Singular value decomposition of the substitution matrix

The singular value decomposition (SVD) method is a standard matrix factorization method [15]. SVD is related to Principal Component Analysis [16]. It has been used in the field of bioinformatics to, e.g., analyze protein sequence alignment score data [17], expression data [18] and position-specific scoring matrices [19].

Any square *N *× *N *symmetric matrix (*N *= 20 for protein substitution matrices) *S *can be expressed as a series of *N *Cartesian vector products ranked according to the decreasing singular values *w*_*K*_:(1)

where  are column *N*-dimensional unit vectors,  are row *N*-dimensional unit vectors (*T *indicates the transpose) and ⊗ represents the Cartesian vector product (the result of which is an *N *× *N *matrix). The vectors  and  form orthonormal sets. The singular values are linked to the square of the Frobenius matrix norm by the following equation:(2)

where *a *and *b *represent amino acids and *s*_*ab *_is the corresponding substitution matrix element. When the series in Eq. 1 is truncated, or some terms are omitted, the remaining terms still yield , a more or less accurate representation of the original matrix *S*.

For a square symmetric matrix either  or . Omitting in Eq. 1 the terms for which , any entry  of the matrix  can be written as a dot product  of the amino acid vectors, where:(3)

where *U*_*Ka *_is the *a - th *component of vector *U*_*K *_and the dimensionality *R *≤ *N *is the number of conserved terms in the series (Eq. 1). By convention, for SVD, the singular values are always positive and ranked by decreasing values. Components for which  correspond to negative eigenvalues, the sign having been "transferred" to one of the singular vectors. This can be easily verified by performing an eigenvalue analysis of the substitution matrix. Notice that SVD and an eigenvalue analysis give the same results for symmetric, square matrices. In Eq 3 we cannot use negative singular values, since this would result in complex amino acid vectors. However, omitting these negative components is also troublesome, since the negative eigenvalues can have large absolute values indicating that their contribution to the matrix is important. This problem is particularly acute for matrices characteristic of short evolutionary distances. For instance, as will be described later, the agreement between the reconstructed matrix and the original is only 28.5% for the PAM10 matrix, whilst it is 94.7% for the PAM500 matrix.

To improve the approximation matrix, we tried the following formula:(4)

where  is an adjustable translation vector and *shift *is an adjustable constant.  and *shift *can be obtained by minimizing the following expression:(5)

From the minimum condition it can be easily shown that the *shift *is linked to  by a relation:

where  is the matrix arithmetic mean (over all its 400 elements) and  is the geometric center of all the amino acid vectors . The components *T*_*L *_of the vector  are obtained by solving a system of linear equations:

where *a*_*L *_is the L-th component of the amino acids vector , *t*_*L *_is the L-th component of the amino acids geometric center  and *N *= 20.

To measure the agreement between  and *S*, we use a quality index (in %) defined as:(6)

**Figure 1:**
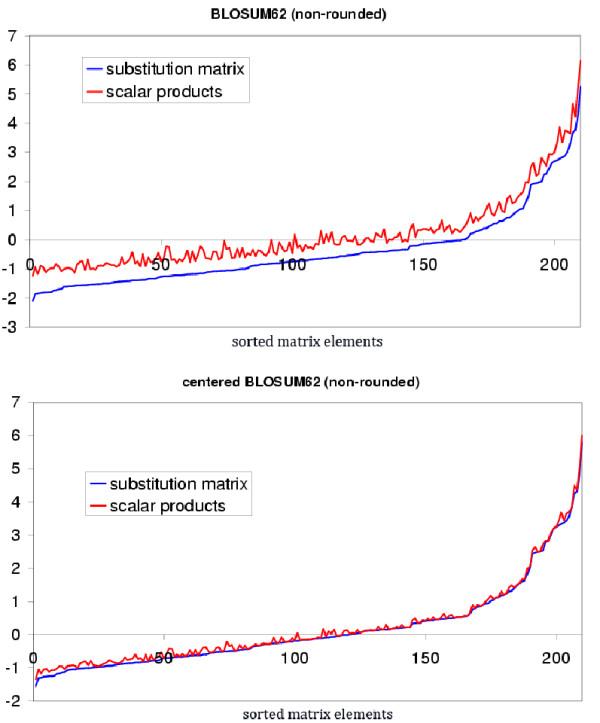
**Top panel: the blue curve is the plot of the substitution matrix elements (210 elements of the lower triangular BLOSUM62, non-rounded, expressed in bit units) sorted by increasing value; the red curve is their approximations, , obtained as the dot products of the *raw, non-centered*, vectors**. Bottom panel: the blue curve is the same as above but with *centered *matrix elements (i.e., the mean of the shifted BLOSUM62 matrix is zero), the red curve is the approximation computed with the *centered vectors*, as described in the text. The x-axis corresponds to the sorted 210 lower triangular matrix elements, e.g., the 210th element is the diagonal element corresponding to the tryptophan, *s*_*WW *_- the largest element in the BLOSUM62 matrix. The y-axis corresponds to the values of the matrix elements. Notice that correlation coefficients are very similar in both cases (0.989 for the curves of the top panel vs 0.998 for the curves of the bottom panel).

We also use the correlation coefficient between the 210 upper triangular entries *s*_*ab *_and the corresponding approximations . Though the correlation coefficient is insensitive to systematic errors (see legend of Fig. 1), it provides a good complementary view.

In order to compare, analyze and visualize the SVD results obtained with different substitution matrices, we need to express them in some standard form. To do that, we center each amino acid vector set, i.e., from each "*raw*" vector set (obtained directly by SVD - Eq. 3) we subtract its geometric mean . If we liken the amino acids to "stars" and the corresponding vectors to their position vectors, the amino acids vector set can be regarded as a "galaxy" and we will herafter refer to it in this way. Each galaxy is characterized by its radius, *R*_*g *_measured as the mean distance of the amino acids from the galaxy center . The different galaxies can then be superimposed by a multidimensional rigid body fit, as described in [20].

Here we need to introduce the concept of "*shifted*" matrices. Shifting a matrix means to add to all matrix elements the same constant. Among all the shifted matrices, we are specially interested in the "*centered*" ones. Centering a matrix consists in subtracting the arithmetic mean of all its 400 elements from each entry, i.e., shifting the matrix of its negative mean.

### Mapping physicochemical properties of amino acids

The Euclidian vector representation of the amino acids allows the mapping of miscellaneous amino acids physicochemical properties into the corresponding multidimensional space. Starting from 28 properties collected in the amino acid index database AAindex [21] and other references we finally kept 17 of them. Nine of them were those of Kidera et al. [6], the others come from various other sources, e.g., [22]. The properties are described in further detail in the Supplementary Table S1 [See Additional file 1].

Amino acids properties are expressed in various scales and units. We thus systematically centered and normalized them. To appreciate the significance of the results, we have compared them with the results obtained for a randomly generated "pseudo-property" (also centered and normalized).

The amino acid physicochemical properties (hydrophobicity, charge, etc.) are scalars, aligned on some line  in the amino acids space. If the substitution matrix were "explained" by a single scalar property, amino acid vectors would have the dimensionality *R *= 1 and the amino acids would all lie on a straight line and should be found with the same order and spread (up to a multiplicative factor) as the property. Of course, this is never the case.

To obtain the best projection of a given property in the amino acids space, it is necessary to find the orientation of the "property axis"  that minimizes the distances between the scaled amino acids properties on  and the corresponding amino acid points in space:(7)

where *a *is an amino acid, *p*_*a *_is the value of the scalar property for amino acid *a *and *λ *is a scaling factor. The condition for the minimum yields for the *K*-th component of :(8)

Where *a*_*K *_is the *K*-th component of vector . Expression  in the above equation is equal to 1 since the properties are centered and normalized.

The "contribution" of a given property to the scoring matrix can be measured as the ratio of the overall spread of the property scalar values *p*_*a *_on the line  to the spread of the amino acid points in space:(9)

This expression, between 0% and 100%, indicates how well the property "explains" the variance of the amino acids in the Euclidian space and thus how much the property *contributes *to the substitution matrix. Notice that the first components of the amino acid vectors represent the best linear approximation of the amino acid spread. Thus, the ratio  (see Eq. 2) represents the upper limit of such a contribution.

## Results and Discussion

### BLOSUM matrices

We studied 16 BLOSUM matrices (from BLOSUM30 to BLOSUM100 by increment of 5, plus BLOSUM62), in their non-rounded form (BLOCKS database: [23]) as well as in their standard form (rounded to integer), usually employed for sequence alignments [See Additional file 2].

Rounding results in some numerical problems so we start the discussion with the results obtained with the original, non-rounded, matrices. We will briefly mention rounding effects afterwards.

The top panel of Fig. 1 shows the result for the non-rounded BLOSUM62 matrix. The entries of the dot products matrix  of the "raw" non-centered vectors (Eq. 3) systematically overestimate the corresponding entries of the original matrix. This difficulty increases with the matrix index, i.e., with decreasing evolutionary distance, as indicated by the quality index (Eq. 6) that decreases from 93.6% for BLOSUM30, through 75.7% for BLOSUM62 to 57.3% for BLOSUM100. However, we observe that the corresponding correlation coefficients between *S *and  are close to one: 0.997, 0.989 and 0.982 respectively. When we repeat the same procedure, starting from the centered matrices, the results are significantly improved (as shown on the bottom panel of Fig. 1). The quality index (and correlation coefficient) vary from 99.6% (0.999) for BLOSUM30 through 98.9% (0.998) for BLOSUM62 to 98.3% (0.997) for BLOSUM100. It is interesting to observe that the dimensionality of the centered matrices is, with very few exceptions, the same as that of non-centered ones. The number of negative eigenvalues remains the same but their absolute value is much smaller for centered matrices. A slightly larger (by about 50%) positive shift of the matrices would even yield quality indices of 100.0% and correlation coefficients of practically 1.000 for all matrices. The latter would, thus, be perfectly represented by dot products. How can we explain such an improvement and what is the effect of the matrix shift on the results?

**Figure 2:**
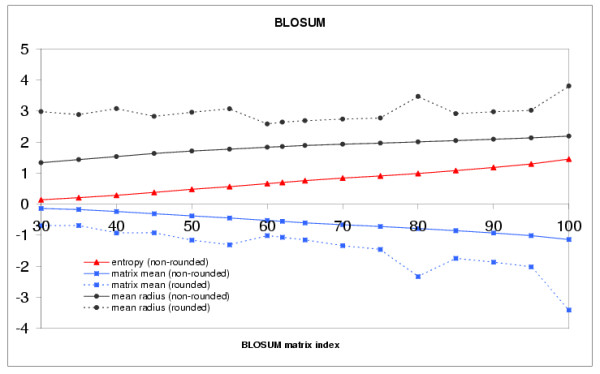
**Plot of the matrix mean (blue), matrix relative entropy (red) and amino acid galaxy radius, *R*_*g *_(black), for the BLOSUM matrix series (solid for rounded and dashed for non-rounded matrices)**. The x-axis corresponds to BLOSUM matrix indices, from 30 to 100 by increment of 5, the y-axis corresponds to the values.

Fig. 2 shows that the matrix mean becomes increasingly negative with increasing BLOSUM indices. We could thus view the BLOSUM matrices as originally centered but undergoing an increasingly negative shift. As mentioned above, it is easy to obtain, even for a moderately positive shift of the matrix, quality indices close to 100%. In the limiting case of a very large positive shift all matrix entries tend to the same value (equal to the shift) and all the corresponding amino acid vectors tend to the same vector, the norm of which is equal to the square root of the shift. On the other hand, for a sufficiently negative shift, we observe that the quality index can decrease to zero. This is the case when, e.g., the shift is such that even the matrix diagonal elements become negative. Negative diagonal elements cannot be represented by the square of vectors and the dot product matrix representation breaks down.

Let us now consider a centered vector set. The corresponding matrix of dot products will be centered, too, and its SVD will yield back the same vector set (possibly rotated). As described earlier, the amino acids can be likened to a "galaxy". If a positive shift is applied to the matrix of dot products, the SVD of this shifted matrix will yield exactly the same galaxy, but having undergone a rotation and a translation in the direction of positive coordinates. The situation is not symmetric when a negative shift is applied to the matrix, since, as mentioned above, the dot product representation is no longer valid when diagonal elements become negative. Thus, the more negative the shift the worse the dot product representation. However, the SVD of a negatively shifted dot products matrix will still yield exactly the same galaxy, except that it cannot be symmetrically translated to the negative coordinates. This causes a systematic overestimation of the matrix entries by the dot products, as is observed in Fig. 1. Let us emphasize that the essential point, here, is the fact that the amino acid galaxy is shift-invariant. It is exactly the same galaxy which yields poor quality indices with the non-centered matrices and much better ones with the centered matrices. This observation led us to represent the approximate matrix  by Eq. 4 above. Omitting  in formula (4) has a limited effect, the *shift *is much more important. With  and *shift *both obtained by the minimization (Eq. 5) above, quality indices for all the substitution matrices analyzed (BLOSUM and PAM) are systematically better than 99.0% and the correlation coefficients better than 0.990. The curves corresponding to *S *and  in Fig. 1 are, in this case, hardly distinguishable.

In any case, our objective in this work is to obtain the best amino acid vectors corresponding to a given substitution matrix, and the centered vectors obtained by SVD fulfill this role perfectly. These centered vectors characterize the amino acids individually, the translation  and the *shift *being the same for all amino acids.

**Figure 3:**
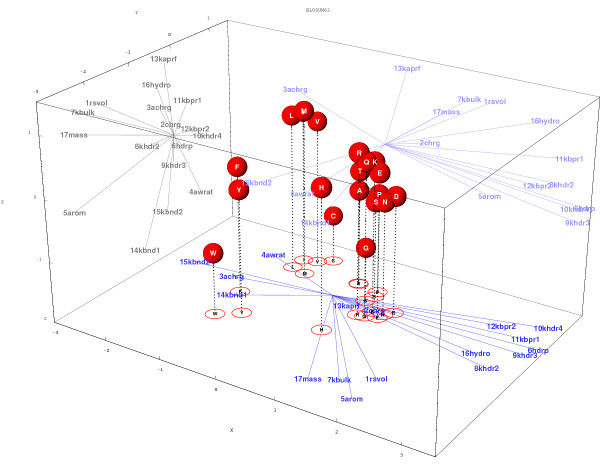
**Three-dimensional projection of the (non-rounded) BLOSUM62 amino acid galaxy together with its physicochemical characteristics**. Property vectors are projected on the left, bottom and rear faces of the parallelepiped. The values on the X, Y, Z axes correspond to the first 3 components of the 20 amino acid vectors.

The galaxy corresponding to the matrix BLOSUM62 is shown in Fig. 3. A movie (animated gif images) showing the evolution of the galaxy for the complete BLOSUM series can be found at the Web address [24] One observes that with increasing matrix indices, the galaxy shows a tendency to swell and gains about 64% in radius (a sort of "little Big Bang" - see Fig. 2 and [24]). However, besides this monotonous, and nearly linear, expansion the global shape of the galaxy does not change much. To analyze the shape variations, we have superimposed all the galaxies, allowing for size scaling. The result (an animated gif image) can also be seen at the previously given Web address. The animation shows that the main, but still not very important, differences occur for matrix indices ≤ 40 (this might correspond to the transition found by Kinjo and Nishikawa [25]). For larger indices, the variations of the galaxy shape are minute. A visual inspection of Fig. 3 reveals that amino acids can be grouped in about six clusters: i) a cluster of aliphatic amino acids: L, I, V, M; ii) a cluster of aromatic amino acids: F, Y, W; iii) a cluster of small, polar or neutral, amino acids: T, S, G, A, P; a cluster grouping positively charged amino acids K, R, negatively charged amino acids E, D and associated amino acids Q, N; v) H and vi) C. To obtain a less subjective clustering we used the k-means algorithm to classify the amino acids into groups using the 20 dimensions. Table 1 shows the results for an increasing number of classes *k*.

**Table 1:** Clustering of amino acids for BLOSUM62 matrix by the k-means algorithm.

2:	C	I	L	M	V	F	W	Y		•			A	G	P	S	T	D	E	N	Q	K	R	H	
3:	F	W	Y	•	C	I	L	M	V	•			A	G	P	S	T	D	E	N	Q	K	R	H	

4:	F	W	Y	•	C	I	L	M	V	•		A	G	P	S	T	•	D	E	N	Q	K	R	H	

5:	F	W	Y	•	C	I	L	M	V	•		A	G	P	S	T	•	D	E	N	Q	K	R	•	H

6:	F	W	Y	•	C	I	L	M	V	•	A	T	•	G	P	S	•	D	E	N	Q	K	R	•	H

The first partition (*k *= 2) separates polar and non-polar amino acids. The next partition separates the latter group into aliphatic and aromatic amino acids. The following partition splits the polar groups into charged (and associated) amino acids and small, polar and neutral, ones. The next partition isolates Histidine from the charged group. Finally the small, non polar and neutral, amino acid group splits into A, T on one side and S, P, G on the other side (the clustering of A and T is noticeable in Fig. 3). The latter splitting is somewhat surprising since one would think that S and T, that are very similar polar residues just differing by a methyl group, would tend to remain together. The amino acid clustering shows only marginal variations with different BLOSUM matrices (data not shown).

We may thus conclude that matrices of the BLOSUM series are characterized by a shift (the negative matrix mean) that increases with the matrix index and an amino acid galaxy with a nearly constant shape, which only shows an expansion with increasing matrix indices. The swelling of the galaxy, i.e., the fact that amino acids are more separated when the matrix index increases, makes sense since for BLOSUM100 the chance of mutating a given amino acid into some other is smaller than the corresponding mutation, for instance, in BLOSUM30.

Two interesting observations are the existence of i) a large correlation (0.977) between the radius of the galaxy, *R*_*g *_and the matrix entropy and ii) a nearly perfect anti-correlation (-0.9999) between the matrix mean and the matrix entropy. As we shall see with the PAM matrices, these observations are not fortuitous and so we defer the discussion regarding these points until the PAM result section.

#### Physicochemical properties

Fig. 3 illustrates the orientation of the physicochemical property vectors, , in the three-dimensional projection of the amino acid space for the BLOSUM62 matrix.

The contributions of the different properties (Eq. 9) to three BLOSUM matrices are summarized in Table 2. Properties that contribute the most to BLOSUM matrices are aromaticity (*5arom*) hydrophobicity (*6hdrp*, *9khdr3*, *10khdr4*), beta-propensity (*11kbpr1*) and, to a lesser extent, properties proportional to the size of the residue: volume (*1rsvol*), bulkiness (*7kbulk*) and molecular mass (*17mass*). It is a little bit surprising to observe that the contribution of the charge is not significantly higher than that of a random property. A possible explanation for this fact is twofold i) the distribution of values for the charge is peculiar: R, K have a charge of +1, D, E have a charge of -1, all other amino acids have a charge of 0 and ii) charged residues cluster with other polar residues (see Fig. 3). It is likely that the conjunction of these two characteristics makes it difficult to find a line representing well the charge property. Aromaticity exhibits a similar value distribution (F, Y and W have a value of 1 and all other residues have a value of 0) but, unlike charged amino acids, aromatic amino acids are well separated from other amino acids in the space. Even though the contributions may seem low, the largest ones are close to the upper limit of about 20% set by . Nevertheless, this indicates that the hydrophobicity (or any other scalar characteristics) represents the "tip of the iceberg" only and thus provides a very partial description of the amino acid properties. The vector representation constitutes a much more comprehensive description.

**Table 2:** Contributions of the physicochemical properties to BLOSUM matrices.

Properties	1rsvol	2chrg	3achrg	4awrat	5arom	6hdrp	7kbulk	8khdr2	9khdr3
BLOSUM30	12.0	5.7	5.4	10.9	13.4	12.0	11.8	12.7	11.7

BLOSUM62	12.7	6.0	7.6	9.6	14.2	17.7	12.1	15.6	17.1

BLOSUM100	12.0	6.4	7.5	8.9	13.1	16.6	11.4	14.7	16.2

									

**Properties**	**10khdr4**	**11kbpr1**	**12kbpr2**	**13kaprf**	**14kbnd1**	**15kbnd2**	**16hydro**	**17mass**	**18rand**

BLOSUM30	11.5	12.3	9.2	5.3	7.2	9.7	10.6	11.6	5.2 ± 1.4

BLOSUM62	17.2	15.5	12.5	5.6	9.0	12.8	13.9	11.6	5.2 ± 1.4

BLOSUM100	16.2	14.9	12.4	5.6	8.8	12.2	13.2	10.8	5.2 ± 1.3

(Contributions are in % see Eq. 9). The property categories are: volume (1rsvol), charge (2chrg, 3achrg), aromaticity (5arom), hydrophobicity (6hdrp, 8khdr2, 9khdr3, 10khdr4, 16hydro), bulkiness (7kbulk), mass (17mass), *α*-propensity (13kaprf), *β*-propensity (11kbpr1, 12kbpr2). The last column 18rand is a simulated "random" property.

Though the values change slightly from one matrix to the other along the BLOSUM series, the above observations remain valid for all matrices.

Other groups have studied the relationship between amino acid properties and various substitution matrices. Tomii and Kanehisa [26] have shown, for instance, that PAM matrix elements are correlated with the volume and hydrophobicity of amino acids. Kinjo and Nishikawa [25] performed an eigenvalue analysis of substitution matrices computed from structure-based alignments for different intervals of sequence similarity. They showed that the first eigenvalue for matrices corresponding to a percentage of sequence similarity larger than 30% is correlated with the relative mutability whereas it is correlated with hydrophobicity below 30%. Our results are consistent with these findings. Notice that we did not consider relative mutability in the physicochemical properties we selected since it bears a direct relationship with the matrix elements.

Substitution matrices are currently used for sequence alignments in their rounded-to-integer form. We observe that the rounding disturbs the results (compare the evolution of the mean and the galaxy radius for the rounded and non-rounded matrices in Fig. 2). The corresponding movie (see the Web page [24]) is also much more chaotic. Thus, although the rounding is probably of no consequence upon the alignments results, one should refrain, when possible, to use rounded matrices to compute amino acid vectors. Notice that the matrix entropy, often provided with the rounded matrices, is in reality that of the non-rounded ones.

### PAM matrices

For the PAM series, unfortunately, we were not able to obtain the original, non-rounded, matrices. We thus analyzed 50 rounded matrices, from PAM10 to PAM500 by increments of 10 [See additional file 3].

As with the BLOSUM series, the difficulty of representing the PAM matrices by the dot products of the "raw" vectors (Eq. 3 increases with decreasing matrix indices (recall that the PAM numbering runs opposite to the BLOSUM numbering, i.e., small indices represent short evolutionary distances). In particular the quality index (Eq. 6) decreases from 94.7% for PAM500 through 70.6% for PAM160 to a mere 28.5% for PAM10. Using the centered matrices, the corresponding values (and the correlation coefficients) are 96.8% (0.985), 89.5% (0.943) and 93.9% (0.923). However, as for the BLOSUM matrices, using the same (centered) vector set and the procedure described in Eq.(5), the quality indices approach 100% and the correlation coefficients is very close to 1.000.

**Figure 4:**
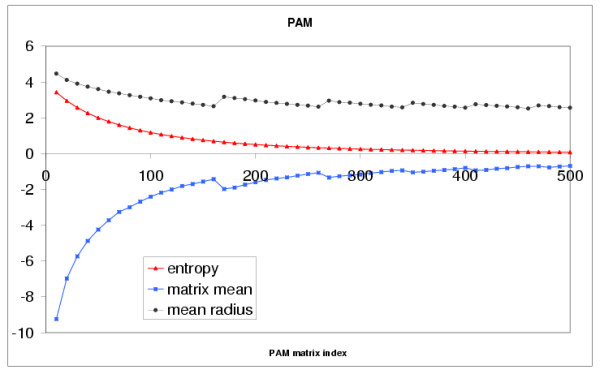
**Plot of the matrix mean (blue), matrix relative entropy (red) and amino acid galaxy radius, *R*_*g *_(black), for the PAM matrix series**. As explained in the text, the observed lack of monotonicity of the matrix mean and galaxy radius curves, is probably due to the fact that rounded PAM matrices were used. The x-axis corresponds to PAM matrix indices, from 10 to 500 by increment of 10, the y-axis corresponds to the values.

As for the BLOSUM series, each PAM matrix is characterized by the matrix shift and the centered amino acid galaxy. The galaxy expands by about 75% from PAM500 to PAM10 (see Fig. 4). Although galaxies corresponding to the PAM matrices have a larger radius than those of the BLOSUM series, they nevertheless bear a close resemblance with the latter. The evolution of the galaxies for the whole PAM series is presented at the Web address [24]. Likewise the BLOSUM series, the shape of the PAM galaxy is quite stable. It is known that there exists a correspondence between the BLOSUM and PAM series. For instance, the EBI Web site [27], gives the following correspondences: PAM100 ↔ BLOSUM90, PAM120 ↔ BLOSUM80, PAM160 ↔ BLOSUM60, PAM200 ↔ BLOSUM52, PAM250 ↔ BLOSUM45. We have scaled and fitted the amino acid galaxies of the PAM matrices to their homologs of the BLOSUM series, and then used a linear interpolation to obtain a kind of "morphing" between the corresponding galaxies. One observes that the corresponding galaxies are very similar, at least in their main features (see [24]). However, having only the rounded PAM matrices, we are not able to discriminate, when differences are observed, which ones are real and which ones are due to the rounding effect. The latter can have a noticeable influence.

Supplementary Table S2 [See Additional file 1] presents the contributions of the physicochemical properties to different PAM matrices. Roughly speaking, we find the same trends that were observed for the BLOSUM series.

#### Relative entropy, galaxy radius and matrix mean

On Fig. 2 the matrix mean, galaxy radius and matrix relative entropy curves for the BLOSUM series appear more or less linear. For the PAM series (see Fig. 4) the corresponding curves, clearly, are not linear. It should be noted, though, that the range of the PAM series is far wider than that of the BLOSUM series since the range BLOSUM90-BLOSUM45 corresponds to the range PAM100-PAM250. Focusing only on the latter range for the PAM series, the curves might very well appear linear. Nevertheless, even if we consider the complete range for the PAM series, there is still a strong anti-correlation, -0.978, between the matrix mean and the relative entropy and a strong correlation, 0.959, between the relative entropy and the galaxy radius. We assume that, without the rounding effect, the calculated correlations would even be better, close to -1, for the anti-correlation between the entropy and the matrix mean, as we observe with the BLOSUM matrices. The relative entropy provided with the substitution matrices is given by the following formula:

where *p*_*a*_, *p*_*b *_are the background probabilities for amino acids *a *and *b *and *q*_*ab *_is the probability of amino acids *a *and *b *to appear in columns of the multiple sequence alignments that were used to compute the substitution matrix. *s*_*ab *_is the matrix element for amino acids *a*, *b*. As described by [1], "M measures the average information available per position to distinguish the alignment from chance". Notice, as pointed out by Bastien et al. [28], that the alignment score between two sequences can be considered as the estimated mutual information between them. It measures how much knowing one of the sequence reduces our uncertainty about the other (more precisely, about the fact that the two sequences are evolutionary related).

The expected score for a given substitution matrix ⟨*s*⟩ = ∑_*a*, *b*_*p*_*a*_*p*_*b*_*s*_*ab *_is negative (otherwise the matrix could not be used to perform local alignments since, on average, it would always be favorable to extend the local alignment) whilst the mutual information is always positive. M is the average score per residue pair when these residues are related by some evolutionary model, whereas ⟨*s*⟩ is the average score for a random model. The mutual information is maximal when amino acids *a *and *b *always covary (see [29]). For this situation we have *M *= *H *= -∑_*a*_*p*_*a*_*log*_2_*p*_*a *_the entropy of the amino acid distribution (corresponding to about 4.2 with the current amino acid probability distribution). The mutual information is minimal (zero) when *q*_*ab *_= *p*_*a*_*p*_*b*_, implying that amino acids are independent.

We observe, for both the PAM and BLOSUM series that the mutual information decreases when the evolutionary distance increases. The interpretation of this fact is that the constraints existing on amino acid mutations become weaker and weaker when the evolutionary distance increases, i.e., it is increasingly probable of replacing an amino acid by any other one. Let us emphasize that this is not to say that amino acid physicochemical properties become irrelevant at large evolutionary distances, but that, in the long run, the protein structure has enough time to adapt locally and accommodate mutations that would be extremely unfavorable at shorter evolutionary distances. In the limiting case, when the mutual information becomes 0, there is no longer any constraint on amino acid mutations and they occur in columns of multiple alignments with a probability equal to the product of their background frequencies. In this regime (known as the twilight zone) sequence alignments do not bring any information regarding a possible homology and other methods, such as fold recognition techniques that take into account the protein structure, should be used [30].

With this picture in mind, the correlation between the galaxy size and the mutual information could be explained as follows: for small evolutionary distances amino acids are far apart in the multidimensional space - they have a definite individuality. When the evolutionary distance increases they get closer to each other - they are less distinguishable, implying that it becomes easier to interchange them. The anti-correlation between the matrix mean and the mutual information is more difficult to explain. We have performed simulations that randomly generated *q*_*ab*_distributions from which we computed pseudo substitution matrices (data not shown). These simulations have shown that there is indeed an anti correlation between the matrix mean and mutual information, although weaker than the one we found for substitution matrices, but we were not able to arrive at a satisfactory explanation of the underlying reason for this observation.

### Distance matrix approach

For the sake of comparison, we also considered the distance matrix approach propounded by a number of groups for obtaining the amino acid vectors. We started with the simple formula:

that is used by a number of authors, e.g., [10]. It should be noted that for the BLOSUM or PAM matrices this distance very often violates the triangle inequality. To obtain the amino acid vectors, we tried several approaches: i) we used the Torgerson's matrix of dot products; ii) we minimized directly the differences between the distance matrix entries and the vector distances; iii) we maximized the negative correlation between the substitution matrix entries and the vector distances. [See Additional file 4].

The third approach seems to be the best in the given context, the results obtained with the Torgerson's matrix approach are the worse. We develop this in more detail in the Supplements. Then we tried the following modified expression:

This distance satisfies the triangle inequality for the BLOSUM matrices and PAM matrices up to index 210. For larger PAM indices it violates the triangle inequality much less than the previous formula (at most 3% of the distance triplets are concerned).

As we show in this paper, it is not necessary to use the distance matrix approach to obtain amino acid vectors. However, if one needs a metric space (see for instance [12]), one can use our amino acid vectors to compute a true metric distance:

### Consensus profile

A possible usage of the amino acid vector representation is for finding the consensus sequence of a multiple sequence alignment. Let us assume that a column of an alignment of three sequences contains the three amino acids: H, R and V. What is the best consensus amino acid for this column? Using amino acid vectors, a straightforward answer to this question exists: it is the amino acid closest to the geometric mean of the amino acids in the column. In the above case it is T.

## Conclusions

Substitution matrices are complex and subtle data structures. They are symmetric, but non-redundant and seem devoid of any simple pattern. Substitution matrices have 210 independent matrix elements, thus, on average, a minimum of about 11 values per amino acid is needed to reconstruct them. This implies that it is illusory to describe amino acids with only two or three characteristics. Our results corroborate this simple reasoning.

Using SVD, we were able to obtain from the substitution matrices a Euclidian vector for each amino acid. This representation is appropriate for a number of analyses that do not rely on sequence comparisons and, instead, need to take into consideration the intrinsic properties of the sequences. As discussed by [12], a metric model of evolution is a prerequisite for the development of fast sequence comparison algorithms. The vector representation we propose allows us to define distances between amino acids that satisfy the three conditions, positivity, symmetry and triangle inequality, defining a Euclidian metric space while avoiding the loss of information incurred when a similarity matrix is converted into a distance matrix. One interesting finding of this work is the fact that substitution matrices of the same series (BLOSUM or PAM) can all be represented by a shift, that only depends on the evolutionary distance, and a set of amino acid vectors (the "galaxy"). Galaxies are very similar but for the radius *R*_*g *_that increases with decreasing evolutionary distances.

Among many other possible applications, the vector representation enables the comparison of different substitution matrices, the calculation of a consensus sequence and the evaluation of the effect of various physicochemical properties in the substitution matrices.

### Software used

Most computations were made with the Scilab software [31]. We used the Microsoft Excel spreadsheet to perform some optimizations, to make some data manipulations and create graphical representations. Mathematica scripts [32] were used to generate the 3D figures. We employed GIMP [33] to create animated gif images used to compare 3D mappings. Programs and data are freely available at [24]

## Authors' contributions

KZ has conceived the project, made the theory, programming and computations. JFG made the biological interpretations and the subsection concerning the matrix entropy (theory, simulations). Both wrote the paper, read and approved the document.

## Supplementary Material

Additional file 1 Supplementary Tables Click here for file

Additional file 2 BLOSUM series amino acid vectors Click here for file

Additional file 3 PAM series amino acid vectors Click here for file

Additional file 4 Supplements Click here for file
