# Supplementary material for: Amino acid "little Big Bang": Representing amino acid substitution matrices as dot products of Euclidian vectors
Source: BMC Bioinformatics. 2010 Jan 4;11:4. doi: 10.1186/1471-2105-11-4 (PMC3098074; doi:10.1186/1471-2105-11-4)
Supplement: Additional file 1 — Supplementary Tables [file 1471-2105-11-4-S1.PDF]

Table 1: Physicochemical properties used in the article.

label	comment	ref	A	C	D	E	F	G	H	I	K	L
1rsvol	residue_volume	1	88.3	112.4	110.8	140.5	189	60	152.6	168.5	175.6	168.5
2chrg	net_charge	2	0	0	-1	-1	0	0	0	0	1	0
3achrg	abs (net_charge)	2	0	0	1	1	0	0	0	0	1	0
4awrat	atomic weight ratio	3	0	2.75	1.38	0.92	0	0.74	0.58	0	0.33	0
5arom	aromaticity	4	0	0	0	0	1	0	0.5	0	0	0
6hdrp	accessibility	5	16	168	-78	-106	189	-13	50	151	-141	145
7kbulk	bulk	6	-1.44	-0.75	-0.54	0.17	1.09	-2.16	0.52	0.21	0.68	0.25
8khdr2	hydrophobicity	6	-0.47	0.06	-0.75	-0.62	1.46	-1.02	-0.46	1.37	-0.16	1.06
9khdr3	hydrophobicity	6	0.11	0.63	-1.74	-1.65	1.24	-0.19	-0.18	0.97	-1.62	1.01
10khdr4	hydrophobicity	6	0.32	1.5	-1.07	-1.03	1.16	-0.03	-0.13	1.52	-1.76	1.14
11kbpr1	beta-propensity	6	-0.51	0.6	-1.17	-1.74	0.88	-0.84	-0.56	1.91	-0.86	0.69
12kbpr2	beta-propensity	6	-0.86	1.14	-1.72	-1.78	0.48	-0.99	-0.1	1.27	-1.19	0.02
13kaprf	alpha-propensity	6	1.35	-0.53	-0.06	1.96	0.37	-1.72	0.59	0.06	0.71	0.93
14kbnd1	bend-structure preference	6	-1.29	1.18	0.74	-1.21	-0.46	1.43	-0.27	-1.3	0.4	-1.36
15kbnd2	bend-structure preference	6	-0.6	-0.19	1.39	-0.27	-0.75	1.73	-0.27	-1.49	0.15	-1.14
16hydro	hydrophobicity	7	2.15	1.2	1.13	1.73	3.46	1.18	2.45	3.88	3.05	4.1
17mass	molar mass	8	89.09	121.16	133.1	147.13	165.19	75.07	155.16	131.17	146.19	131.17

label	comment	ref	M	N	P	Q	R	S	T	V	W	Y
1rsvol	residue_volume	1	162.2	125.1	122.2	148.7	181.2	88.7	118.2	141.4	227	193
2chrg	net_charge	2	0	0	0	0	1	0	0	0	0	0
3achrg	abs (net_charge)	2	0	0	0	0	1	0	0	0	0	0
4awrat	atomic weight ratio	3	0	1.33	0.39	0.89	0.65	1.42	0.71	0	0.13	0.2
5arom	aromaticity	4	0	0	0	0	0	0	0	0	1	1
6hdrp	accessibility	5	124	-74	-20	-73	-70	-70	-38	123	145	53
7kbulk	bulk	6	0.44	-0.34	-0.71	0.22	1.16	-1.21	-0.67	-0.34	2.08	1.34
8khdr2	hydrophobicity	6	0.2	-1.25	0.9	-1.24	-0.57	-1.19	-0.97	0.42	2.06	1.16
9khdr3	hydrophobicity	6	0.72	-0.6	0.21	-0.46	-1.52	-0.33	0.01	0.77	1.55	1.04
10khdr4	hydrophobicity	6	1	-0.96	-0.72	-1.05	-1.07	-0.46	-0.36	1.38	0.67	-0.07
11kbpr1	beta-propensity	6	0.45	-1	-1.26	0.19	-0.28	-0.54	0.57	1.84	0.61	1.02
12kbpr2	beta-propensity	6	0.24	-1.19	0.86	-0.42	-0.13	0.22	0.86	1.66	0.42	1.21
13kaprf	alpha-propensity	6	1.39	-0.97	-1.72	0.57	-0.16	-0.99	-0.68	-0.09	0.23	-1.25
14kbnd1	bend-structure preference	6	-1.24	1.19	1.03	-0.14	0.28	0.74	0.11	-1.63	0.83	0.94
15kbnd2	bend-structure preference	6	-1.29	1.27	1.98	-0.12	-0.03	1.02	0.14	-1.32	-0.52	0.3
16hydro	hydrophobicity	7	3.43	1.05	3.1	1.65	2.23	1.4	2.25	3.38	4.11	2.81
17mass	molar mass	8	149.21	132.12	115.13	146.15	174.2	105.09	119.12	117.15	204.23	181.19

column ref:

1: AA-index(http://www.genome.jp/aaindex/),DBGET index: H GOLD730102
2: http://www.imb-jena.de/IMAGE/aa/amino_acid.txt
3: http://www.expasy.ch/tools/pscale/Ratioside.html
4: our own definition
5: AA-index (http://www.genome.jp/aaindex/), DBGET index: BIOV880101
6: Table III (page 39) of A.Kidera et al., Journal of Protein Chemistry, Vol.4, No 1, p.23-54 (1985)
7: http://www.imb-jena.de/IMAGE_AA
8: http://fr.wikipedia.org/wiki/Acide_aminé#Liste_des_principaux_acides_aminées

Table 2: Contributions (in % Eq. 7 of the article) of the physicochemical properties to PAM matrices. The property categories are: volume (1rsvol), charge (2chrg, 3achrg), aromaticity (5arom), hydrophobicity (6hdrp, 8khdr2, 9khdr3, 10khdr4, 16hydro), bulkiness (7kbulk), mass (17mass), α-propensity (13kaprf), β-propensity (11kbpr1, 12kbpr2). The last column 18rand is a simulated "random" property.

Properties	1rsvol	2chrg	3achrg	4awrat	5arom	6hdrp	7kbulk	8khdr2	9khdr3
PAM500	22.2	4.7	5.6	16.1	27.3	20.7	21.4	23.1	21.1
PAM160	17.2	7.5	6.2	11.9	18.3	18.0	16.5	17.4	18.0
PAM10	13.8	9.3	4.9	8.9	14.4	14.2	13.3	13.1	13.5

Properties	10khdr4	11kbpr1	12kbpr2	13kaprf	14kbnd1	15kbnd2	16hydro	17mass	18rand
PAM500	15.1	17.0	14.0	4.3	9.2	10.7	19.0	20.7	5.1±2.4
PAM160	16.5	15.9	13.8	5.4	10.4	12.2	16.4	15.7	5.2±1.8
PAM10	12.9	12.5	12.2	5.4	9.4	10.1	12.4	12.6	5.2±1.5
