# Supplementary material for: Amino acid "little Big Bang": Representing amino acid substitution matrices as dot products of Euclidian vectors
Source: BMC Bioinformatics. 2010 Jan 4;11:4. doi: 10.1186/1471-2105-11-4 (PMC3098074; doi:10.1186/1471-2105-11-4)

SUPPLEMENTS

Distance approaches

The substitution matrix is not a distance matrix since there exist negative entries and a non-zero diagonal. To be able to treat it as a distance matrix, it should be first converted to a new matrix with the required properties. As explained in the paper, we have tried two methods to compute the distance:

$$d_{ab} = s_{aa} + s_{bb} - 2s_{ab} \tag{1}$$

and

$$d_{ab}^2 = s_{aa} + s_{bb} - 2s_{ab} \tag{2}$$

where s_{aa}, s_{bb}, s_{ab} are the entries of the substitution matrix. Both formulas violate the triangle inequality, though the second one seems much better. For example, the first formula generates 22 violations (over 3420 triangles tested) for BLOSUM30 (standard integer-rounded series), 0 for BLOSUM62 and BLOSUM100. The second formula does not generate any violation for the whole BLOSUM series. For the PAM series the difference is more striking: the first formula is responsible of 336 violations for PAM500, 63 for PAM160, 14 for PAM10. With the second formula these numbers are: 56 for PAM500, 0 for PAM160 and PAM10. The triangle inequality is violated only for PAM indices ≥ 210.

To obtain the vectors from the distance matrix (violating or not the triangle inequality), we have tested and compared three approaches:

1. The "royal way" of distance matrix analysis [1, 2] converts the distance matrix into the *Torgerson*'s matrix of scalar products from which, by the SVD method, it is possible to obtain the vectors. The dimensionality of the vectors is given by the SVD, as described in the paper.

2. Direct minimization of the squared norm of the difference matrix:

$$\min_{\vec{a}, \vec{b} \dots} \sum_{a,b} (d_{ab} - \| \vec{b} - \vec{a} \|)^2 \tag{3}$$

   One starts from one-dimensional amino acid vectors (i.e. scalars) $\vec{a}, \vec{b}, \dots$ and increase the vector dimensionality until the vector distances $\| \vec{b} - \vec{a} \|$ are sufficiently close to the distance matrix elements d_{ab}.

3. Bypassing the need of a conversion, following the simple idea that high substitution matrix elements correspond to short distances, low or negative ones to long distances. This yields another approach – maximization of the negative correlation coefficient between the 190 non-diagonal elements of the substitution matrix and the vector distance matrix:

$$\max_{\vec{a}, \vec{b} \dots} (-c(S, D)) \tag{4}$$

where $c(S, D)$ is the correlation coefficient between the 190 non-diagonal elements of the substitution matrix S and the vector distance matrix D, $d_{ab} = \| \overrightarrow{b} - \overrightarrow{a} \|$. As in the second method above, the dimensionality of the vectors $\overrightarrow{a}$, $\overrightarrow{b}$ ... is progressively increased until the (negative) correlation coefficient is sufficiently high.

The weak point of the first two methods is the conversion to distances, Eq.1 and 2 above. If the diagonal matrix elements are not sufficiently large, the distances (and even the squared distances) can be negative. Another problem is that the formulas (1, 2) are not necessarily the best, nor the only possible.

The quality of these approximations can be measured, for the first two methods, by the quality index (Eq. 4 in the paper) calculated with the 190 non-diagonal elements of the distance matrix D (Eq.1 and 2) and the vector distances matrix D and by the correlation coefficient between these two data sets. With the third method one uses the centered and normalized values of S and D to calculate the correlation.

The three methods are illustrated on Fig. 1 for the "most problematic" matrix PAM500 (see the paper). The results shown are representative of the whole set of substitution matrices. The Torgerson matrix approach (top panel) provides the worst results, the "correlation approach" (bottom panel) is clearly the best. Passing by the Torgerson matrix is mathematically elegant, but when the distance matrix entries are not all exactly Euclidean distances, to approach the Torgerson matrix is not the same problem as to approach the distance matrix. This is why the second method performs better. The only problem of the third method seems to be that the resulting vectors are defined only on a relative scale. We thus recommend it, in particular for the cases when the starting data are not really Euclidian distances.

Coding of the "property vectors" in the substitution matrices

The $\overrightarrow{P}$ vectors (Eq. 5-7 of the paper) describe how a given physico-chemical property "contributes" to the substitution matrix. A related question is whether, and to which measure, any particular amino acid physicochemical property is "coded" or "can be found" in the substitution matrix. This question can be formulated as follows: Is there a line $\overrightarrow{Q}$ such that the scaled projections of the amino acids on this line, $\lambda \overrightarrow{a} \overrightarrow{Q}$, coincide with the property values p_a on this line? The orientation of the line $\overrightarrow{Q}$ can be found by solving the following minimization problem:

$$\min_{\overrightarrow{Q}} \sum_a (p_a - \lambda \overrightarrow{a}.\overrightarrow{Q})^2 \tag{5}$$

It can easily be shown that the R components of the vector $\overrightarrow{Q}$ must be a solution of the linear system

$$\lambda \sum_K^R (\sum_a a_I a_K) Q_K = \sum_a p_a a_I, \ I = 1 \ldots R, \tag{6}$$

where a_I is the I-th component of the amino acid vector $\overrightarrow{a}$, Q_K is the K-th component of the normalized vector $\overrightarrow{Q}$, R is the dimensionality of the space and λ is a scaling factor.

The figure of merit of this operation can be measured either by the ratio

$$\frac{\sum_a (\lambda \overrightarrow{a}\overrightarrow{Q})^2}{\sum_a p_a^2} = \sum_a (\lambda \overrightarrow{a}\overrightarrow{Q})^2 \tag{7}$$

(note that the values p_a are centered and normalized) or by the correlation coefficient between the property p_a and the projections $\overrightarrow{a}\overrightarrow{Q}$.

It should ne noted that neither $\overrightarrow{P}$ nor $\overrightarrow{Q}$ are principal axes of the amino acid galaxy and that they are not necessarily parallel.

We were rather surprized by the results obtained with Eq. 5. It yields always values close to 100% (Eq. 7). Even the "random property" exhibits a value of 89% (BLOSUM30) and the lowest value observed was 85% for *3achrg* (BLOSUM30). These results would lead to the conclusion that any "property" can always be "found" in the substitution matrix, which is rather puzzling. However, straightforward tests with randomly generated "amino acids" and "properties" show that this surprising result is due to the fact that the dimensionality of the amino acids space is equal (or very close) to the number of amino acids. If the space dimensionality is reduced, the ratio (7) decreases rapidly. We must thus conclude that the question posed at the beginning of this paragraph cannot be formulated in the above terms.

References

[1] T. Foucart. *L'analyse des donnes (Mode d'emploi)*. Dunod, Paris, 1997.

[2] http://forrest.psych.unc.edu/teaching/p230/torgerson.pdf.

Figure 1: Top panel: Torgerson method, middle panel: difference matrix minimization, bottom panel: anti correlation maximization method.

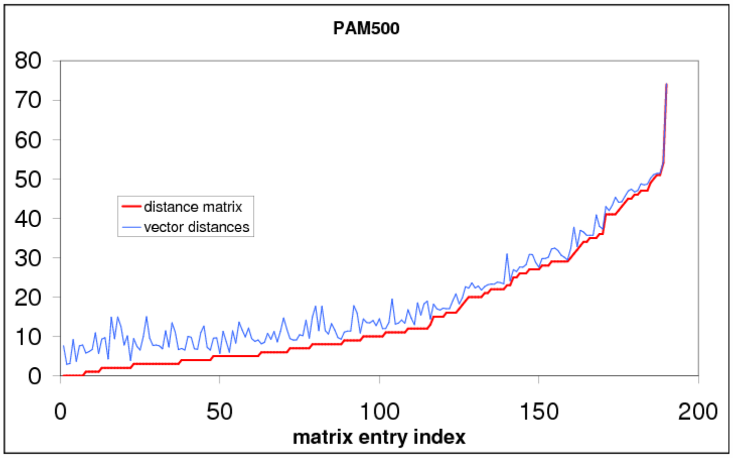

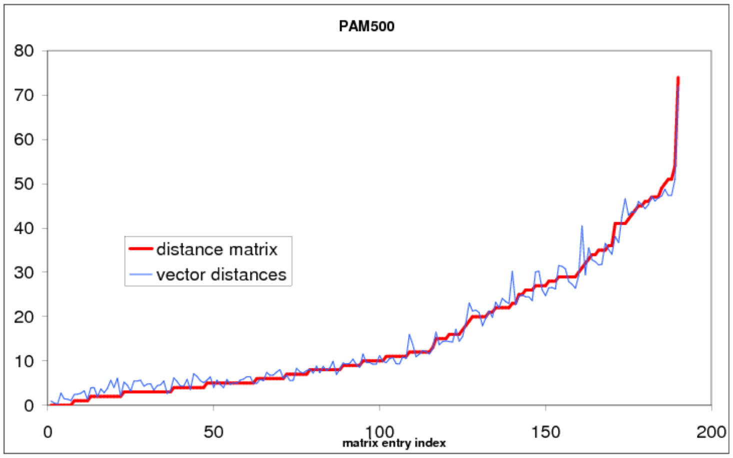

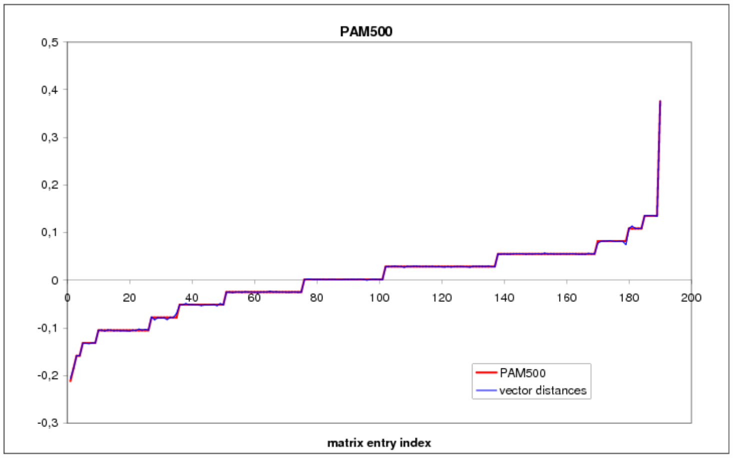

Supplement: Additional file 4 — Supplements [file 1471-2105-11-4-S4.PDF]
